# Diaphragmatic paralysis in a neonate with circumferential skin creases Kunze type

**DOI:** 10.1002/mgg3.2003

**Published:** 2022-06-23

**Authors:** Gao Chun Fang, Ding Kaiwei, Zeng Lingkong, Tao Xuwei

**Affiliations:** ^1^ Department of Neonatology Wuhan Children's Hospital of Tongji Medical College, Huazhong University of Science & Technology Wuhan China

**Keywords:** circumferential skin creases‐Kunze type, diaphragmatic paralysis, *TUBB*

## Abstract

**Background:**

A range of clinical features have been confirmed with heterozygous mutations in Beta Tubulin (*TUBB*), including skin creases, facial deformities, abnormal cerebral structures, and intellectual disability, and were defined as Circumferential Skin Creases Kunze type (CSC‐KT).

**Methods:**

Clinical information was obtained retrospectively on a neonate hospitalized in the Neonatal Intensive Care Unit, Wuhan Children’s Hospital. Genomic DNA was extracted from circulating leukocytes of the proband according to standard procedures.

**Results:**

The neonate presented dyspnea resulting from diaphragmatic paralysis, accompanied by other typical features of CSC‐KT. Additionally, exome sequencing confirmed a new variant (NM_178,014. 4: c. 1114 A > G) in *TUBB*. We also summarized features described in previous cases, thus representing phenotype extension of CSC‐KT.

**Conclusion:**

Our report is the youngest confirmed case, which could extend the current phenotype of CSC‐KT as well as the clinical diagnostic approach.

## Graphical abstract

We report a case of neonatal onset of CSC‐KT with dyspnea due to l diaphragmatic paralysis, extending a new phenotype and genotype for this genetic disease.

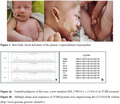

## 1 INTRODUCTION

Circumferential skin creases‐Kunze type (CSC‐KT) is a rare autosomal‐dominant inherited disease associated with pathogenic variants of *TUBULIN BETA* (*TUBB*; OMIM: #191130) and *MICROTUBULE‐ASSOCIATED PROTEIN*, *RP/EB FAMILY*, *MEMBER 2* (*MAPRE2*; OMIM: #605789) (Tinsa et al., 2009; Wouters et al., 2011). The typical clinical manifestations are skin creases on limbs, also known as Michelin Tire baby syndrome (Ross, 1969). Wouters et al. (2011) coined the term “CSC‐KT,” specifically referring to children with multiple malformations such as cleft palate, facial deformity, growth retardation, genital malformation, and intellectual disability (Kunze & Riehm, 1982; Wouters et al., 2011) in addition to CSC.

*TUBB* and *MAPRE2* gene mutations are associated with CSC‐KT, which are consistent with the genetic heterogeneity of the disease (Isrie et al., 2015). The clinical phenotype was reported prior to the widespread clinical application of gene sequencing technology. A published limited cohort study found that *TUBB* and *MAPRE2* genes were associated with the genotype–phenotype profile of CSC‐KT (Breuss et al., 2012). The authors reported three missense mutations and one nonsense mutation in the calci‐protein homology domain of the *MAPRE2* gene and three missense mutations in the *TUBB* gene (Goldspink et al., 2013). Pathogenic mutations in the *MAPRE2* and *TUBB* genes cause either an altered affinity of *MAPRE2* to microtubules or defects in *TUBB*’s assembly of tubulin heterodimers; both can lead to developmental disorders in the central nervous system, face, and skin (Isrie et al., 2015).

The *TUBB* gene is widely expressed in different mammalian tissues, particularly in the central nervous system during the development and growing skin (Wawro et al., 2017). Class I β‐tubulin is highly expressed in neuronal progenitor cells, postmitotic neurons during fetal brain development, and human fibroblasts (Fallet‐Bianco et al., 2014; Romaniello et al., 2018). Unlike the α‐tubulin proteins, β‐tubulin forms dynamic heterodimers to form microtubules (Dent & Gertler, 2003; Jaglin & Chelly, 2009). Microtubules are critical parts of the cytoskeleton and are involved in intracellular transport, essential for mitotic chromosome separation and cell migration during embryonic development (Breuss et al., 2017).

Patients with *TUBB* variants exhibit neurologic features, including microcephaly, dysplasia of the cerebellum and basal ganglia, and corpus callosum agenesis, in addition to the congenital symmetrically skin folds (Madrigal et al., 2019). *MAPRE2* encodes a microtubule‐associated protein—an essential regulator of microtubule dynamics and recombination during cell differentiation (Goldspink et al., 2013; Isrie et al., 2015).

The tubulin‐coding complexity is reflected by the tubulin composition diversity, including 10 types of β‐tubulin and 12 types of α‐tubulin proteins, in addition to the multiplicity of chemical properties (Kawauchi, 2017). For example, interactions with Microtubule‐Associated Proteins and tubulin Post‐Translational Modification enzymes regulate microtubule dynamics, neuronal polarity, cell motility, and intracellular transport (Park & Roll‐Mecak, 2018; Vemu et al., 2017). A study by Sferra et al. (2020) revealed that intracellular vesicle transport of both, the Epidermal Growth Factor and Transferrin, were modified in fibroblasts from patients with *TUBB* mutations, suggesting that the mutations in the *TUBB* gene impaired microtubule function and dynamics, and confirming that *TUBB* plays a vital role in microtubule‐dependent vesicle transport. Further, in vitro evidence also suggested that microtubules work in concert with actin to establish and maintain spatial and temporal coordination of cell migration (Sferra et al., 2020). The correct migration of the neural crest to the branchial arch is critical during the development of palatal facial microtubules. CSC‐KT’s typical facial features are associated with fetal tubulin dysfunction, resulting in neural crest cell migration disorders.

Ross reported the first case in 1969 based on clinical features of CSC (Ross, 1969). Subsequently, there were a few intermittent case reports with the first genetically diagnosed case in 2015 (Isrie et al., 2015). The patient we present here had neonatal‐onset, except for his typical CSC, facial dysmorphism, hypospadias, and severe gastroesophageal reflux. The baby presented with dyspnea. In addition, a new mutation was detected in the *TUBB* gene. Our results may expand the phenotype of diseases related to *TUBB* abnormalities.

## 2 MATERIALS AND METHODS

### 2.1 Ethical compliance

This study was reviewed and approved by the Ethics Committee of Wuhan Children's Hospital, Tongji Medical College, Huazhong University of Science and Technology (2021R050‐E01), and the written informed consent was obtained from the legal guardian.

### 2.2 Clinical evaluation and genetic analysis

The patient was evaluated at the Wuhan Children's Hospital, and all the clinical data were obtained in a retrospective manner. Genomic DNA samples were isolated from whole blood samples using a Wizard® Genomic DNA Purification Kit (Promega, USA) according to the manufacturer’s protocol. Full exon sequencing was performed by automated Sanger sequencing. RefSeq gene accession number NM_178014.4 for the *TUBB* gene was used. The sequencing results were analyzed using the BLAST program (http://www.ncbi.nlm.nih.gov/blast).

## 3 CASE REPORT

### 3.1 Case presentation

The patient was a 7‐day‐old boy admitted with dyspnea for 7 days. The baby was G1P1, G40^+6^W, vaginal delivered, the Apgar Score was 9 in 1 min and 10 in 5 min, birth weight was 2.8 kg (P15, −1.2 *SD*), head circumference was 34 cm (P50, 0 *SD*), and length was 50 cm (P15, −0.1 *SD*). Ten minutes after birth, the baby developed shortness of breath, moaning, and dyspnea and was then urgently treated with ventilation in other hospital. Without any improvement after 7 days, the boy was transferred to our ward.

### 3.2 Physical examination on admission

**FIGURE 1:**
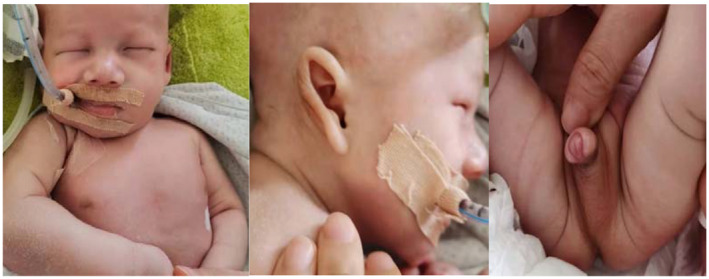
Skin folds, facial deformity of the patient, cryptorchidism, hypospadias

The body temperature was 36.5°C, heart rate was 152 beats/min, and blood pressure was 72/50 mmHg. Abnormal findings on examination included: skin folds on the shoulders, arms, wrists, and lower limbs, epicanthal folds, low‐set and posteriorly rotated ears with overfolded thick helices, widely spaced nipples, hypospadias, and undescended testicles (Figure 1). Other physical examinations showed that clear consciousness, bilateral pupils' sizes, and light reactions were normal; breathing was steady under assisted ventilation; and auscultations of both lungs were symmetrical. A continuous murmur was audible throughout all phases of the cardiac cycle. The abdomen was soft, and bowel sounds were normal. There was free movement of all limbs with normal muscle force and muscle strength.

### 3.3 Supplementary examinations

**FIGURE 2:**
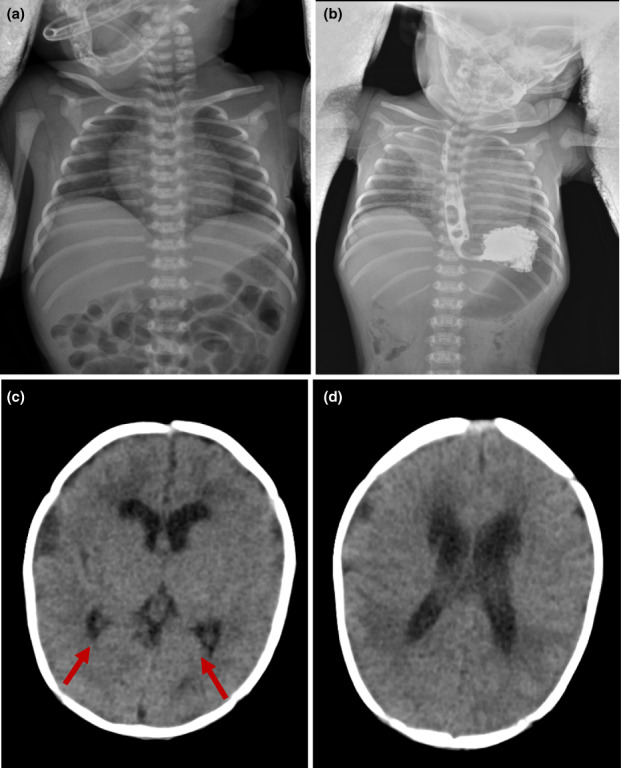
(a) Chest radiographs: Bilateral diaphragmatic lifts. (b) Gastrointestinal angiography: Unobstructed esophagus, gastroesophageal reflux. (c) Head‐CT: Bilateral subependymal cysts. (d) Head‐CT: Bilateral lateral ventricles slightly enlarged

**FIGURE 3:**
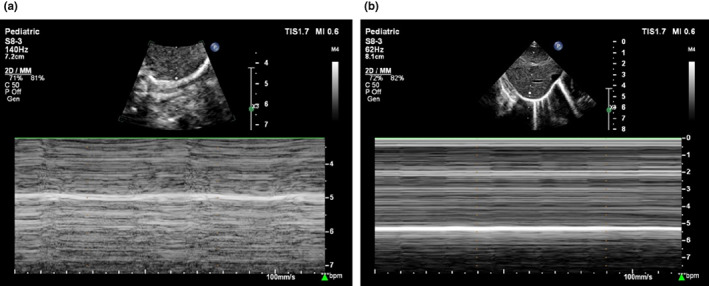
Diaphragm ultrasound: Deduced bilateral diaphragmatic activity, (a) Left side, (b) Right side

There were no obvious abnormalities in the routine blood count, the biochemical parameters, and the blood amino acid and urine organic acid screening. The Torch‐IgM, blood, and sputum cultures were negative. Results of electrocardiogram and electrocorticography were normal, and an atrial septal defect (about 4.6 mm) was detected by Echocardiography. Fiberoptic bronchoscopy showed no abnormalities in the larynx, trachea, and bronchus. Chest radiographs suggested enhanced lung textures and bilateral lifting of the diaphragm (Figure 2a). The gastrointestinal angiography indicated an unobstructed esophagus with gastroesophageal reflux (Figure 2b). Head‐CT scan revealed enlarged bilateral lateral ventricles, paraventricular hypodensity, and subependymal cysts (Figure 2c,d), without any deformities in the corpus callosum, vermis cerebelli, and brainstem. Diaphragm ultrasound showed decreased diaphragm activity (Figure 3), indicating bilateral diaphragm palsy.

### 3.4 Treatment and outcome

Mechanical ventilation and supportive treatments were given during hospitalization. Several attempts were made to detach from the respirator, and the child immediately developed severe dyspnea such as chest breathing and passive diaphragm lifting (Video S1). The suspected reason was the bilateral diaphragmatic paralysis and the relative reduction of the thoracic space. Moreover, the backflow of stomach contents into the lungs caused by severe gastroesophageal reflux also could lead to decreased respiratory function. The parents refused the recommendation of diaphragmatic plication, abandoned all treatments after 20 days of hospitalization, and the baby soon died.

### 3.5 Genetic analysis

**FIGURE 4:**
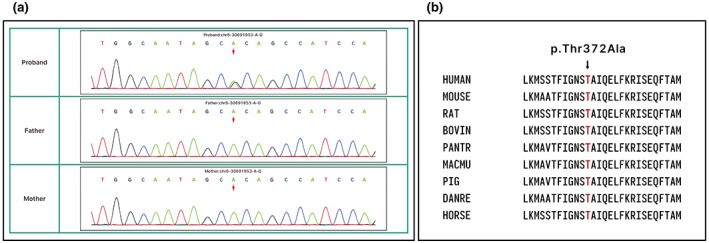
(a) Familial pedigrees of this case, a new mutation NM_178014.4:c.1114A > G in *TUBB* occurred. (b) Multiple amino acid sequences of TUBB proteins were aligned using the CLUSTALW website (http://www.genome.jp/tools/clustalw/)

Genomic DNA was extracted from circulating leukocytes, and a heterozygous missense genomic variant, NM 178014.4. c.1114A > G, resulting in a substitution p. (Thr372Ala), was discovered in the *TUBB* (Figure 4). Parental DNA analysis revealed that the variant was de novo, not present in either parent and was not present in the GME Variome database. The affected threonine at position 372 is located in the N‐terminal domain of the *TUBB* and is highly conserved in vertebrates and invertebrates homologs.

## 4 DISCUSSION

Eleven cases of CSC‐KT were confirmed by gene sequencing from 2015 to 2020 (Dentici et al., 2018; Feng et al., 2020; Isrie et al., 2015; Li et al., 2020; Niu et al., 2020) including six cases of the *TUBB* and five cases of the *MAPRE2* variants. Previous studies have shown that two patients with homozygous pathogenic variants in *MAPRE2* had severe neurological dysfunction, accompanied by intellectual impairment and seizures, whereas there was no neurological dysfunction in the patients with pathogenic *TUBB* variants. Case 3 (Li et al., 2020) was *TUBB* mutation‐positive (NM_178014) C.925C > G, p. Arg309Gly), in which the clinical phenotype was the transposition of great arteries, L3–L4 hemicone causing scoliosis, and speech expression disorder. The child had some characteristics of the two clinical syndromes associated with the *TUBB* mutation but did not conform to either clinical diagnosis. This broad clinical phenotype suggests that *TUBB* is present in multiple organs during multiple developmental processes. Case 7 (Dentici et al., 2018) is a 9‐year‐old boy, who was genetically sequenced with a novel complex heterozygous mutation at the *TUBB*‐N terminus (NM_001293212)c.218 T > C, p.(Met73Thr) from his mother, who had only facial deformations and surrounding skin wrinkles. The clinical phenotype was microcephaly, facial deformity, severe mental developmental delay, seizures, cerebral cortex atrophy, corpus callosum dysplasia, and other neurological disorders. This is the first reported case of the CSC‐KT caused by heterozygous mutation of the *TUBB* gene N‐terminus. Significant clinical variations were identified, and the role of region‐specific mutations in the pathogenesis of CSC‐KT was confirmed (Dentici et al., 2018). Clinical features of the present family and published patients with *MAPRE2* and *TUBB* mutations were summarized in Table 1.

**TABLE 1:** Clinical features of the present family and published patients with *MAPRE2* and *TUBB* mutations

Case	Country of origin	Time	Age	Mutation		Inheritance	Parental affected?	Parental consanguinity	Ceases	Facial deformities	Neurological	Others
Our case	China	2021	7 d	*TUBB*	c.1114A > G, p. (Thr372Ala)	Heterozygous, de novo	No	No	Limbs, shoulders, neck, arms, wrists, and lower limbs	Low set dysmorphic ears and broad nasal bridge	Bilateral lateral ventricles slightly enlarged, bilateral subependymal cysts	Hypospadias, wide spaced nipples, gastroesophageal reflux, diaphragmatic paralysis, atrial septal defect
Niu et al	China	2020	11 m		c.227 T > A, p. (Val76Asp)	de novo	No	No	Limbs, shoulder, and trunk	Epicanthal folds, cyrturanus, and ocular hypertelorism	Cortical dysplasia, corpus callosum hypoplasia, dysmorphic basal ganglia and thalamus, hypoplasia of the cerebellum and brainstem, and widening of the extracerebral space. Intellectual disability	Developmental retardation
Li et al	American	2020	5 y		c.925C > G, p. (Arg309Gly)	de novo	No	No	No	Cleft palate	Minor logopathy	Transposition of the great arteries, deformity of l3‐L4 hemicone
Isrie et al	Turkey	2015	18 m		c.665A > T, p. (Tyr222Phe)	de novo	No	Yes	Limbs	Low set dysmorphic ears, broad nasal bridge, plat face, epicanthal folds, blepharoptosis, ocular hypertelorism	–	Wide spaced nipples, 2nd and 3rd toe syndactyly
Isrie et al	Norway	2015	5 y		c.43C > A, p. (Gln15Lys)	de novo	No	No	Limbs and neck, disappeared at 5 y old expect the wrists	Cleft palate, low set dysmorphic ears, broad nasal bridge, plat face, blepharoptosis	–	Short neck, tapering fingers
Isrie et al	Canada	2015	15 y		c.43C > A, p. (Gln15Lys)	de novo	No	No	Limbs, fingers, neck, and penis	Cleft palate, low‐set dysmorphic ears, broad nasal bridge, microphthalmia, outer canthus folds, microstomia	–	Wide spaced nipples
Dentici et al	Italia	2018	9 y		c.218 T > C, p. (Met73Thr)	Heterozygous, maternally inherited	Mother: only facial deformity and surrounding skin wrinkles	No	Minor folds in limbs and neck	Low‐set dysmorphic ears and broad nasal bridge	Intellectual disability and growth retardation	Gastroesophageal reflux, atrial septal defect; perpetuate the left superior vena cava
Isrie et al	Spain	2015	15 m	*MARPE2*	c.203A > G, p. (Asn68Ser)	homozygous, parents are heterozygous carrier	Father: minor folds in infant	Yes	Limbs and neck	Cleft palate, Low‐set dysmorphic ears, broad nasal bridge, and blepharophimosis, plat face	–	–
Isrie et al	Tunisia	2015	19 y		c.260A > G, p. (Tyr87Cys)	Homozygous, parents DNA not available	No	Yes	Limbs, improved but visible	Cleft palate, low‐set dysmorphic ears, broad nasal bridge, plat face, microphthalmia, and Epicanthal folds, ocular hypertelorism	–	Hypospadias, short neck, 2nd and 3rd toe syndactyly
Isrie et al	Belgium	2015	8 y		c.427C > T, p. (Arg143Cys)	Heterozygous, de novo	No	No	Limbs, disappeared in 4 y	Low hairline, broad nasal bridge, plat face, microphthalmia, Epicanthal folds, microstomia, blepharoptosis, micrognathia	–	Hypospadias
Isrie et al	Belgium	2015	6 y		c.454C > T, p. (Gln152^a^)	Heterozygous, maternally inherited	No	No	Limbs, spontaneous improved	Cleft palate, broad nasal bridge, blepharophimosis, plat face, microphthalmia, microstomia, micrognathia	–	The fifth fingers clinodactyly
Feng et al	China	2019	2 y		c.518G > A, p. (Arg173Gln)	de novo	No	No	Symmetric circumferential skin creases on forearms and ankles	Low‐set dysmorphic ears, broad nasal bridge, and plat face	Growth retardation, logopathy, bilateral lateral ventricles slightly enlarged	The second to fourth toes of both feet have partial syndactyly like malformation while normal foot

^a^ Uncertain amino acid type.

The case we report here was the first genetic‐confirmed CSC‐KT in the neonatal period. The genotype of *TUBB* (NM_178014. 4) c. 1114 A > G, p.(Thr372Ala), is a new variant that occurred de novo in our proband. The predominant clinical manifestation was dyspnea caused by bilateral diaphragmatic paralysis, accompanied by typical CSC, facial dysmorphism, hypospadias, cryptorchidism, subependymal cyst, and severe gastroesophageal reflux. It is possible to conjecture that the reason might be that the *TUBB*‐gene mutation damages the function and dynamics of microtubules, which further affects the cooperative work of fibroblast microtubules and actin resulting in functional abnormalities of corresponding muscles.

Neonatal diaphragmatic paralysis is a rare cause of respiratory distress, traumatic delivery with cervical hyperextension, and/or chest surgery causing phrenic nerve damage (Gerard‐Castaing et al., 2019). Birth injury mainly causes unilateral diaphragm injury and is accompanied by ipsilateral brachial plexus injury, resulting in ipsilateral upper limb dyskinesia. Our patient had no related medical history, and the muscle strength and tension of limbs were normal. Given this, we hypothesize that diaphragmatic paralysis was highly related to microtubule dysfunction due to abnormal expression of the C‐terminal variant in the *TUBB*.

## 5 CONCLUSION

Our report extends the phenotype of CSC‐KT and suggests that *TUBB* variants may cause structural or/and functional abnormalities in multiple tissues or/and organs. Therefore, it is necessary to expand clinical thinking in treating children with suspicious phenotypes. In addition, genetic testing can prevent undiagnosed or misdiagnosed cases.

## CONFLICT OF INTEREST

The authors declare that they have no competing interests

## AUTHORS’ CONTRIBUTION

Gao Chun Fang and Ding Kaiwei collected the clinical data and drafted the initial manuscript. Zeng Lingkong interpreted the result and reviewed the manuscript. Tao Xuwei drafted, reviewed, and revised the manuscript. All authors agreed to accept responsibility for this work and agreed to the final manuscript as submitted.

## ETHICS STATEMENT

This study was approved by the Human Research Ethics Committee of the Wuhan Women and Children Medical Care Center (2021R050‐E01). The written consent was obtained from the parents of the neonate.

## Supporting information

Video S1

## Data Availability

The data that support the findings of this study are available from the corresponding author upon reasonable request.
